# 3D Heteronuclear Magnetization Transfers for the Establishment of Secondary Structures in SARS-CoV-2-Derived RNAs

**DOI:** 10.1021/jacs.1c01914

**Published:** 2021-03-30

**Authors:** Jihyun Kim, Mihajlo Novakovic, Sundaresan Jayanthi, Adonis Lupulescu, Eriks Kupce, J. Tassilo Grün, Klara Mertinkus, Andreas Oxenfarth, Christian Richter, Robbin Schnieders, Julia Wirmer-Bartoschek, Harald Schwalbe, Lucio Frydman

**Affiliations:** † Department of Chemical and Biological Physics, Weizmann Institute of Science, Rehovot 7610001, Israel; ‡ Department of Physics, Indian Institute of Space Science and Technology, Valiamala, Thiruvananthapuram 695 547, Kerala, India; ∥ Bruker Ltd, Banner Lane, Coventry CV4 9GH, United Kingdom; ⊥ Institute of Organic Chemistry and Chemical Biology, Center for Biomolecular Magnetic Resonance, Johann Wolfgang Goethe-University, D-60438 Frankfurt/Main, Germany; § Aleea Nicolae Titulescu nr. 8, Turda, 407405 Judeţul Cluj, Romania

## Abstract

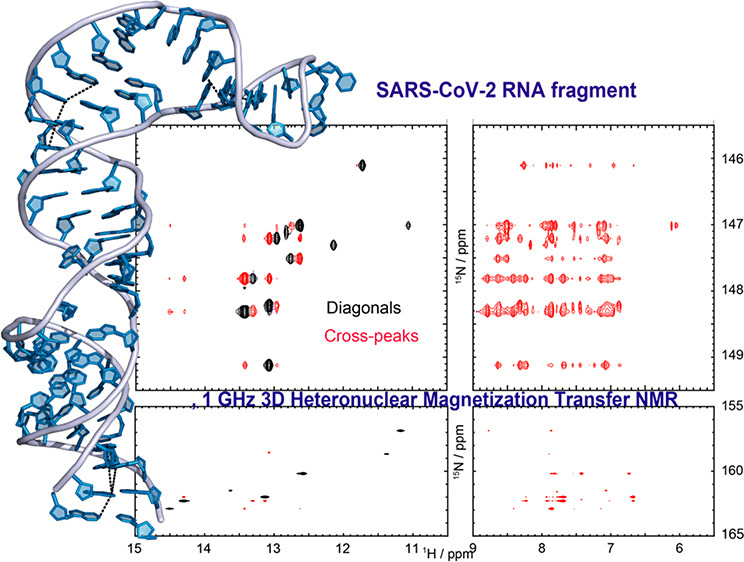

Multidimensional NOESY experiments targeting correlations between exchangeable imino and amino protons provide valuable information about base pairing in nucleic acids. It has been recently shown that the sensitivity of homonuclear correlations involving RNA’s labile imino protons can be significantly enhanced, by exploiting the repolarization brought about by solvent exchanges. Homonuclear correlations, however, are of limited spectral resolution, and usually incapable of tackling relatively large homopolymers with repeating structures like RNAs. This study presents a heteronuclear-resolved version of those NOESY experiments, in which magnetization transfers between the aqueous solvent and the nucleic acid protons are controlled by selecting specific chemical shift combinations of a coupled ^1^H–^15^N spin pair. This selective control effectively leads to a pseudo-3D version of HSQC-NOESY, but with cross-peaks enhanced by ∼2–5× as compared with conventional 2D NOESY counterparts. The enhanced signal sensitivity as well as access to both ^15^N–^1^H and ^1^H–^1^H NOESY dimensions can greatly facilitate RNA assignments and secondary structure determinations, as demonstrated here with the analysis of genome fragments derived from the SARS-CoV-2 virus.

RNAs fulfill numerous essential roles, including the propagation of genetic information, the regulation of expression, and support for protein synthesis.^1−4^ Underlying this functional diversity is an equally diverse set of structures, of conformational dynamics, and of interactions with proteins, other nucleic acids, ligands, and ions, which NMR can explore with exquisite detail at physiological conditions.^5−12^ Especially valuable in such analyses are the spectral signatures of the nitrogen-bound protons, which combine chemical shift resolution with valuable information about base pairing. NMR on RNAs thus usually starts with investigations of the imino and amino protons.^13−15^ Imino resonances in particular appear at substantial downfield chemical shifts, well resolved from other ^1^H peaks, and thereby facilitating RNA structural and binding studies.^7,16,17^ Conspiring against a more widespread use of these resonances is their labile nature, as chemical exchanges with water broaden these peaks and complicate their observation.^18,19^ Particularly hurt by chemical exchanges is the transfer of structurally relevant NOE information from the iminos/aminos to neighboring protons, a problem that further compounds the notoriously low signal-to-noise ratio (SNR) of NOESY cross-peaks.

Recently, we have proposed time-domain (L-PROSY^20^) and frequency-selective magnetization transfers (HMT^21^) methods that can bypass these complications, and substantially shorten imino- and amino-based RNA 2D NOESY experiments. As RNA constructs become larger, however, 2D homonuclear correlations become insufficient to distinguish all the protons involved. The chemically shifted attached nitrogens open the possibility to distinguish among these peaks—for instance, among guanosine and uracil imino resonances—making NOESY experiments involving heteronuclear editing/separation a valuable tool in RNA elucidations.^22^ These experiments, however, are even more sensitivity-handicapped than their 2D homonuclear counterparts, since the additional heteronuclear transfer steps that they involve are also often compromised by the rapid imino↔water chemical exchanges.^23^ Many resonances thus remain undetected in these experiments, or fail to generate NOESY cross peaks. The present study presents a way to alleviate this handicap based on what we denote as HETeronuclear MAgnetization Transfer (HETMAT) NOESY; a pseudo-3D NMR experiment making up for the aforementioned losses at the expense of readily available *a priori* information. HETMAT’s substantial sensitivity enhancements of the ensuing ^15^N–^1^H–^1^H correlations over conventional counterparts are shown here with structural elucidations from sizable fragments taken from the SARS-CoV-2 genome.

Figure 1a illustrates the idea proposed for recording these pseudo-3D ^15^N–^1^H-resolved NOESY correlations. The experiment starts by assuming that the ^15^N–^1^H frequency pairs in the system are *a priori* known—for instance, from a preliminary heteronuclear 2D correlation—and that the positions of all such pairs are sufficiently resolved to be individually identified. Following the principles introduced in the HMT experiment,^21^ the aim then is to selectively saturate or invert the ^1^H peak associated with each such individual ^15^N–^1^H pair, so as to allow fast exchanges between these hydrogens and the solvent to reinstate the full magnitude of the NOE (or if inserting an isotropic mixing period, of the TOCSY) cross-peaks. Arraying such 1D experiment for every resolved heteronuclear ^15^N–^1^H spin pair then leads to a 3D information that is akin to that arising from an HSQC-NOESY (or HSQC-TOCSY)—but without suffering from the effects of fast exchanges with the solvent. With this as a guide, different approaches were assessed to selectively perturb a proton based on predefined ^15^N–^1^H frequency pairs. Selective versions of BIRD^24^ and TANGO^25^ were considered, yet the best performance for the desired 2D spectral manipulation was found in selective, longitudinal cross-polarization (CP) experiments.^26,27^ Heteronuclear CP is normally used as a broadband technique in solids and liquids to transfer magnetizations between ^1^H and X nuclei, as mediated by either *J* or dipolar couplings. However, narrowband versions of CP that selectively excite specific spin pairs according to their resonance offset have also been demonstrated.^28−30^ In the present work, we relied on narrowband CP not to transfer polarization, but rather to selectively *invert*^1^H magnetizations for specific combinations of ^1^H and ^15^N offsets, i.e., as a first step in the HETMAT sequence. To perform this, simultaneous ^1^H/^15^N radiofrequency (RF) fields were applied on-resonance to an *a priori* selected imino group, while fulfilling the ω_1H_ = ω_1N_ = ω_1_ ≤ 2*πJ*_NH_ Hartmann–Hahn matching condition.^31^ Unlike a conventional CP, this does not spin-lock a transverse proton magnetization, but rather nutates it in a subspace composed from single (H) and two-spin (HN) operators.^32^ Assuming that the RF fields are applied along the *x-*axes of the spins’ doubly rotating frames, it can be shown (see Supporting Information) that the evolution starting from an initial *H*_*z*_ polarization is then
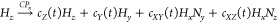
1where the {*c*}_*i*=*Z*, *Y*, *XY*, *XZ*_ describe the time-dependent coefficients for each spin operator. To maximize the subsequent Overhauser cross-relaxation of a proton thus excited, we seek to achieve an inversion—or at least the largest possible perturbation away from equilibrium—of the initial *H*_*z*_. Restricting for concreteness the discussion to on-resonance Hartmann–Hahn matching conditions, the relevant dynamics are then
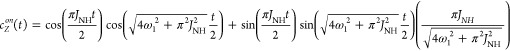
2(Other coefficients and comparisons are given in the Supporting Information (SI)). To evaluate the Overhauser effects arising from such an inversion, a second, reference scan is also acquired, where Hartmann–Hahn matching conditions are no longer fulfilled. To do so, the ^15^N offset is shifted far off-resonance (Figure 1b); *H*_*Z*_’s time-dependence then becomes
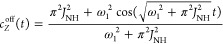
3

**Figure 1:**
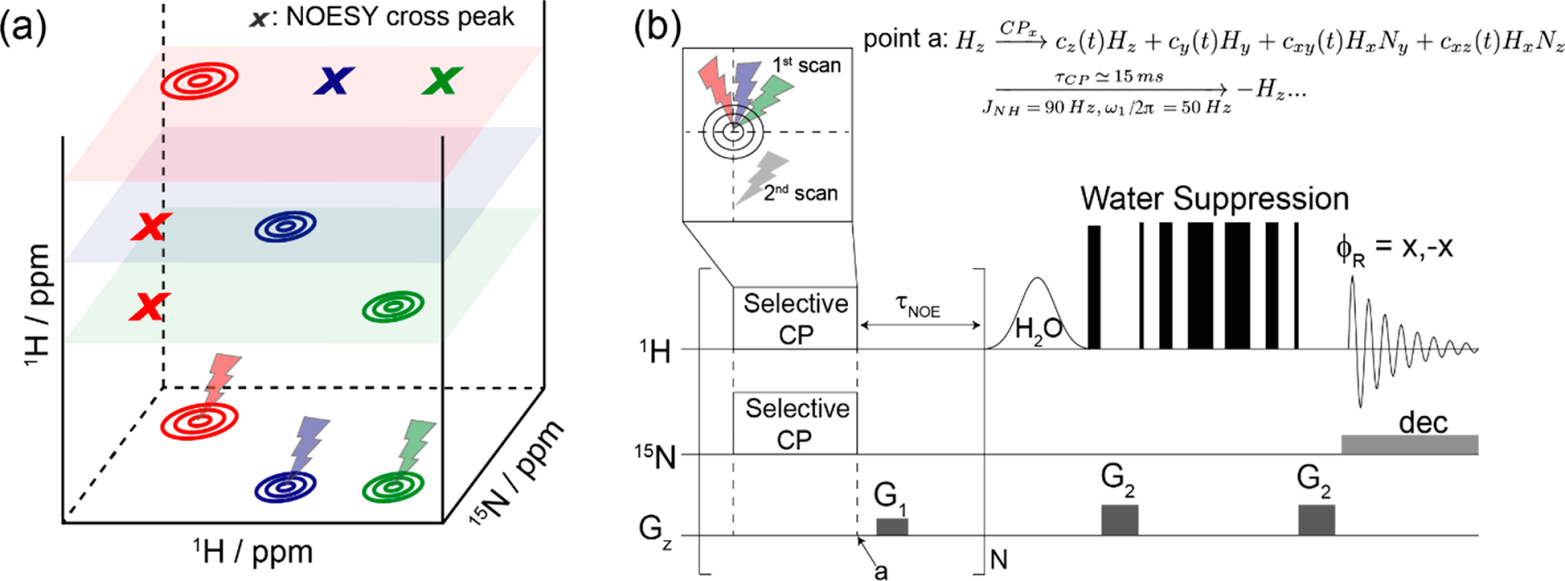
(a) Schematic representation of HETeronuclear MAgnetization Transfer (HETMAT), proposed for detecting HSQC-NOESY-type 3D correlations on labile sites. The experiment maps the NOE cross-peaks associated with a given ^15^N–^1^H spin pair along a third ^1^H shift dimension, using 2D-frequency-specific saturations of the imino groups in the heteronuclear correlation plane. (b) Scheme proposed for executing HETMAT, based on looping selective cross-polarization (CP) modules that perturb the proton longitudinal magnetization for a specific ^15^N–^1^H frequency pair, leaving all other ^1^Hs untouched. The selective longitudinal CP and an NOE mixing (*τ*_NOE_) period are repeated *n* times to enhance the NOE cross-peaks by exchanges with the solvent, and the full spectrum is acquired with a scheme that suppresses the water signal. The selective CP is applied either on- or off-resonance in consecutive scans and data evaluated after receiver phase cycling (*ϕ*_R_ = *x*, −*x*) in order to isolate the NOEs; “dec” indicates the ^15^N decoupling used during the acquisition (see Supporting Information for additional details).

With these expressions for *c*_*Z*_^on^(*t*) and *c*_*Z*_^off^(*t*) coefficients for a given *J*_NH_, it is possible to find optimal ω_1_ and mixing time *τ*_CP_ values that maximize their absolute difference—and hence the magnitude of the subsequent homonuclear NOE. For certain cases like ω_1_/2π = 2*J*_NH_, this can be done analytically: *τ*_CP_ = 1/*πJ*_NH_ will then lead to optimal *c*_*Z*_^on^ = −1, *c*_*Z*_^off^ = +1 conditions. Spectral selectivity, however, generally demands working with ω_1_/2π ≤ *J*_NH_. Given prototypical *J*_NH_ = 90 Hz values, we chose working with ω_1_/2π = 50 Hz; for these conditions, eqs 2 and 3 predict a *τ*_CP_ = 16 ms for maximizing |*c*_*Z*_^on^ – *c*_*Z*_^off^|. This is in good agreement with the *τ*_CP_ = 15 ms measured experimentally on RNAs for achieving maximal HETMAT NOEs (SI Figure S2). SI Figures S3 and S4 further analyze the inversion efficiency and the chemical shift selectivity achieved under these conditions, showing that sizable |*c*_*Z*_^on^ – *c*_*Z*_^off^| > 1 perturbations can be achieved, and that spectral resolutions of ca. ± 50 and ±25 Hz then characterize the ^1^H and ^15^N dimensions, respectively. This selectivity sufficed for targeting our SARS-CoV-2-derived RNA fragments when studied at 1 GHz (23.5 T), but led to some peak cross-talk when examined at 600 MHz (SI Figure S4). The sensitivity with which this selective CP subtraction provided its ^1^H–^15^N correlations was comparable to or larger than that arising from conventional HSQC or HMQC experiments (SI Figure S4a); while higher CP fields ω_1_ could increase the sensitivity further, this would be achieved at the expense of sacrificing spectral resolution. These efficient, selective heteronuclear inversions were then looped as previously described,^17,18^ for the sake of enhancing the iminos’ NOE correlations.

The method described above was employed in the analysis of two SARS-CoV-2-derived RNA fragments, seeking to introduce heteronuclear resolution in imino–imino and imino–amino ^1^H–^1^H NOE correlations. As peak assignments for the smallest of the targeted fragments, the 5_SL5b+c domain of SARS-CoV-2’s RNA, have been reported,^33^ experiments were collected on this fragment mainly for sensitivity comparisons. All the diagonal and cross-peaks in the fragment’s ^15^N–^1^H HSQC and ^1^H–^1^H NOESY spectra are clearly identified by HETMAT spectra acquired at 1 GHz (Figure 2). Besides the additional spectral dimension available in HETMAT NOESY thanks to the heteronuclear separation, significant SNR enhancements are evidenced when this experiment is compared against conventional 2D counterparts of the same duration (Figure 2, on top of each panel; notice that since conventional 3D HSQC-NOESY acquisitions on this sub-mM sample would take days to complete, 2D HMQC-NOESY versions of the experiment were used in this comparison). Note as well that several imino–imino correlations—including those between U8-G22, U8-U23, U6-G20, and G24-G26 (purple arrows and fonts in the spectra)—are only detected by HETMAT NOESY. As some of these distances exceed 5 Å, the possibility that they reflect spin-diffusion effects cannot be discarded. Additional examples of HETMAT’s sensitivity advantage over its HMQC-based counterpart for this sample are presented in SI Figure S5.

**Figure 2:**
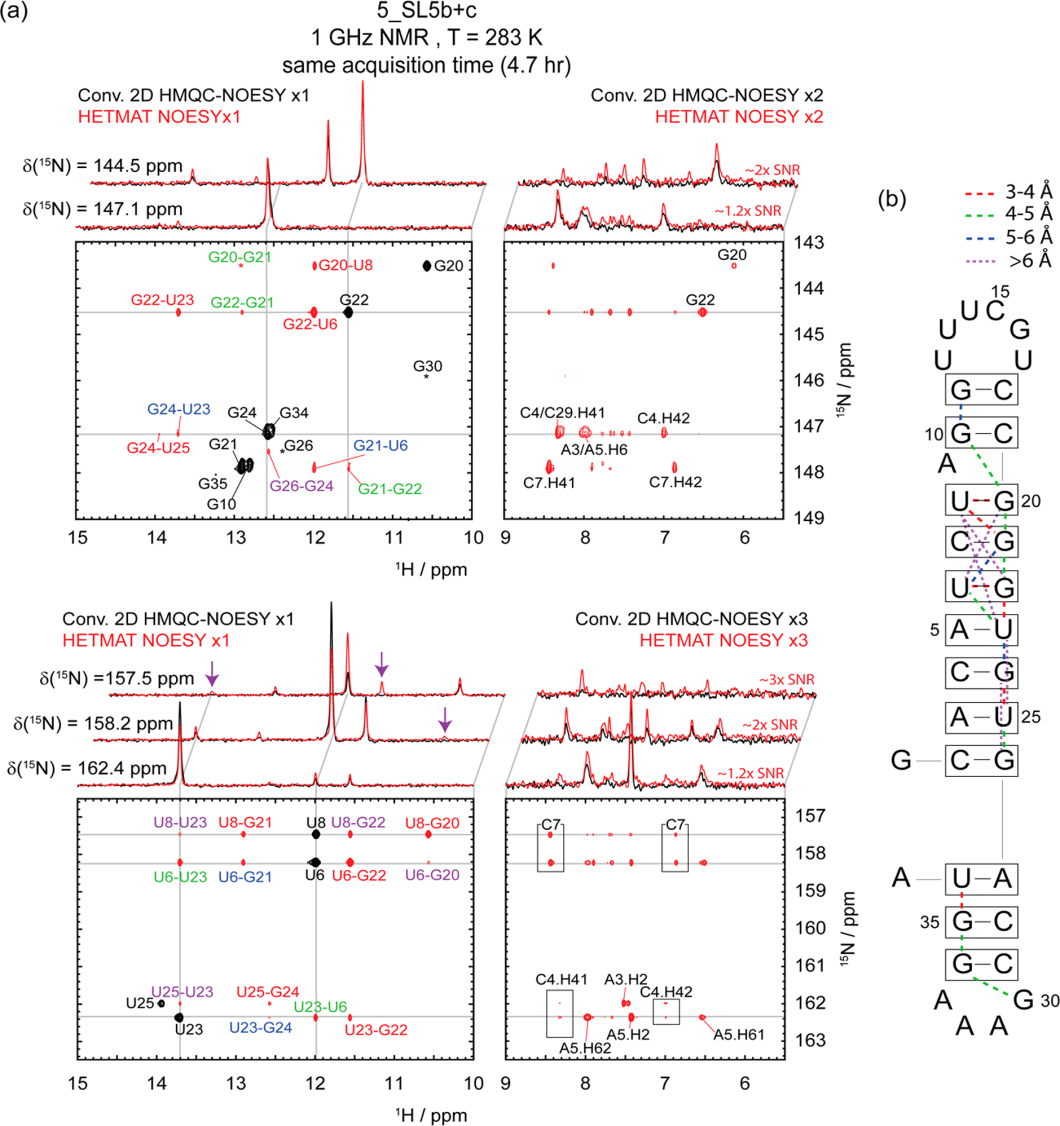
(a) HETMAT and 2D HMQC NOESY data showing imino–imino and imino–amino proton correlations for the SARS-CoV-2-derived 5_SL5b+c RNA fragment in (b), measured at 1 GHz and 283 K. To facilitate viewing of the 3D HETMAT data, contours were projected into the ^15^N–^1^H plane, with black and red contours used to represent the NOESY diagonal and cross-peaks, respectively. Assignments reported for these diagonal and cross-peaks are annotated in red, green, blue, and purple according to the predicted distances; among the peaks labeled in purple, G26-G24, U8-U23, U8-G22, and U6-G20 are cross-peak correlations observed in the HETMAT NOESY but not in the HMQC-NOESY experiment. Shown on top for comparison are 1D slices extracted at the indicated ^15^N shifts from conventional 2D HMQC-NOESY (175 ms mixing, black) and HETMAT NOESY (red), collected on the same sample using identical acquisition times. For the HETMAT NOESY acquisitions, RF fields *ω*_1_/2π = 75 Hz with 20 loops and a τ_NOE_ = 30 ms for mixing were used for the faster exchanging G35 residue; for all the rest, a CP with ω_1_/2π = 50 Hz and 7 loops with τ_NOE_ = 125 ms mixing were used. (b) Secondary structure of 5_SL5b+c; dashed lines denote the correlations observed by HETMAT between specific base pairs, color-coded according to their estimated distances. See SI Figure S11 for a 3D rendering of the indicated connectivities.

The performance of the HETMAT NOESY experiment is further illustrated in Figure 3, for the larger SARS-CoV-2-derived 5_SL8 RNA fragment. In this case, 2D HMQC-NOESY cannot resolve all the proximate peaks even at 1 GHz—for instance, the traces arising from δ(^15^N) = 148.2 and 148.3 ppm—leading to the multiple identical NOE cross-peak patterns due to overlapping signal contributions. By introducing a heteronuclear dimension, HETMAT can help identify the individual NOE correlations for each imino proton, while enjoying a substantial gain in sensitivity. Furthermore, as illustrated and exploited by the results in Figures 2 and 3, HETMET’s focusing on one specific residue per scan enables tailoring both the heteronuclear CP fields as well as the details of the NOE mixing, to the chemical and spectral nature of the residue being tackled. Better-resolved residues can thus be studied with higher RFs leading to shorter CPs, while correlations to broad sites characterized by enhanced solvent exchanges can be studied using more loops and shorter *τ*_NOE_s. Additional comparisons between HETMAT and HMQC-based experiments for this sample are shown in SI Figures S6 and S7. The latter highlights results obtained at a lower field, where resolution becomes more limited for closely positioned peaks.

**Figure 3:**
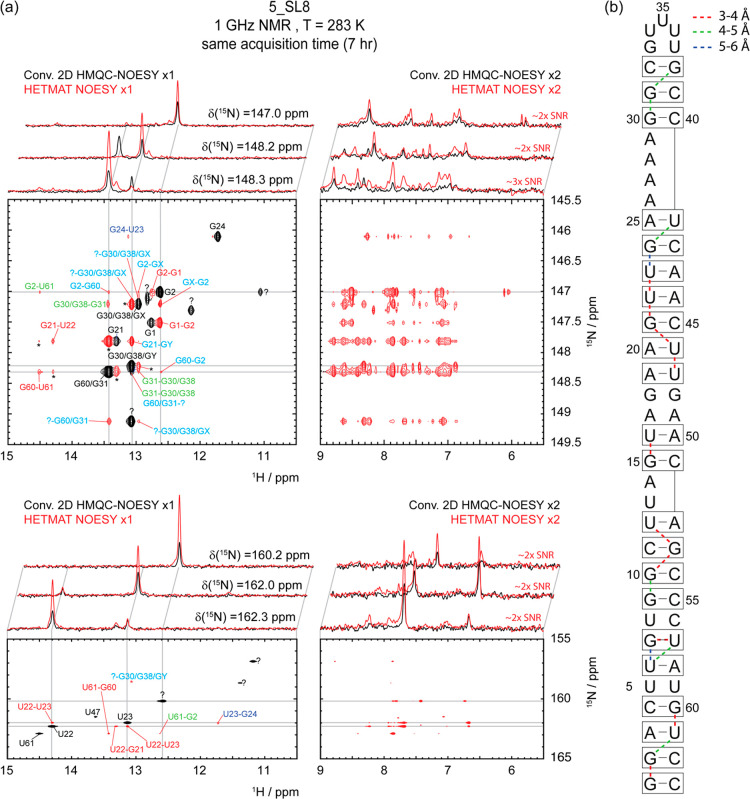
(a,b) Idem as in Figure 2, but for the larger 5_SL8 fragment shown on the right. The color-code used here is akin to that in Figure 2, except that diagonal peaks with uncertain assignments are labeled with black question (“?”) marks, and ambiguous off-diagonal counterparts are marked by question marks, or by GX, GY labels. Newly observed NOE correlations are labeled in cyan. Asterisks indicate artificial signals arising due to insufficient selectivity; 1D slices from conventional HMQC-NOESY (150 ms mixing) and HETMAT NOESY are shown on top. For the broader peaks, an RF CP field ω_1_/2π = 75 Hz with 17 loops and *τ*_NOE_ = 50 ms mixing was used; otherwise, CP with ω_1_/2π = 50 Hz and 10 loops with *τ*_NOE_ = 80 ms mixing were used. Notice that at this field HETMAT NOESY has sufficient selectivity to resolve peaks with very similar ^15^N chemical shifts (e.g., δ(^15^N) = 148.2 and 148.3 ppm, traces on top); this ability decreased at lower fields (SI Figure S7). The apparent difference in resolution between the guanosine and uridine peaks (top and bottom contours) reflects the different chemical shift ranges plotted along F_1_ in each case. See SI Figure S11 for a 3D rendering of this structure, showing the indicated connectivities.

Despite the selectivity provided by the combination of narrowband CP and the use of high magnetic fields, 5_SL8 is a case where total site resolution is not feasible even at 1 GHz. SI Figure S8 exemplifies this for a region where the selective CP for a residue denoted as peak 1, simultaneously labeling a second residue peak 2. This notwithstanding, and as a result of the cross-talk’s asymmetric behavior between the peaks, unambiguous NOE correlations end up becoming achievable by comparing the HETMAT spectra for peaks 1 and 2. This is typical for complex RNA structures such as this one, where differential solvent exchange rates of the individual iminos involved in the cross-relaxation can end up breaking the symmetry of otherwise identical RF manipulations. Peaks marked by asterisks in Figure 3 illustrate other instances where the narrowband CP was insufficiently selective to separate closely spaced ^15^N–^1^H peaks, yet where similar analyses as in Figure S8 allowed us to identify genuine NOE correlations originating from these groups. In this respect, it is worth noting that the rates of solvent exchange and the relaxation properties of the protons may limit HETMAT’s potential gains: the experiment’s sensitivity will usually be larger when magnetization is transferred from fast-exchanging to slow-exchanging protons. This can explain why certain cross-correlations fail to show up, even if involving base pairs in adjacent positions. While this makes certain peak assignments still ambiguous, HETMAT’s improved resolution and sensitivity gains allowed us to establish a number of imino proton correlations that had not been previously reported.^33^

In summary, a novel experiment that can enhance the sensitivity and resolution of homonuclear NOESY correlations by targeting selected ^1^H–^15^N spin pairs was introduced for targeting labile protons. The resolution of the resulting experiment—particularly at high fields—ended up comparable to that of 3D acquisitions. This was achieved by using selective longitudinal CP for incorporating the heteronuclear information: by avoiding reliance on coherent polarization transfers to/from the heteronucleus, this route also helped increase the overall sensitivity. Selective CP requires *a priori* knowledge of the 2D ^15^N–^1^H correlations; in return, it enables customizing the saturation/inversion conditions, mixing time and number of loops *N*, to each individual residue. As solvent exchanges vary widely among NHs, and as they can be assessed *a priori* by the broadness of the targeted ^1^H peak, this customization helps maximize the NOE enhancements. This selectivity also helped enhance the effective resolution among overlapping sites, using asymmetric buildup considerations. HETMAT’s sensitivity gains can also enable studies at higher, physiologically relevant temperatures, despite the increase in chemical exchange rates. This is illustrated in SI Figure S9, which shows that although both HETMAT- and HMQC-based NOESYs suffer upon increasing temperatures as a result of faster exchanges, HETMAT can still deliver a very similar information content as obtained at lower temperatures. This opens interesting possibilities to investigate RNA structures at physiological conditions. Another intriguing possibility exists upon working under mismatched Hartman-Hahn conditions (Figure S10), which according to simulations and experiments could increase the NOE-derived cross-peaks about 50% compared to matched CP conditions, while allowing lower ω_1_ fields and thus better spectral selectivity. It also remains to be seen to what extent these gains associated with frequency-domain manipulations can be preserved, as spectral crowding in the heteronuclear plane increases.

## Supporting Information Available

Experimental section; Time evolution of an I_*z*_ state upon establishing longitudinal cross-polarization on- and off- the Hartmann–Hahn conditions; HETMAT’s inversion efficiency, ^15^N–^1^H spectral selectivity and sensitivity vs HMQC-NOESY on SARS-CoV-2 fragments; Improving HETMAT’s spectral selectivity; HETMAT vs HMQC-NOESY performances at room temperature; HETMAT’s improved sensitivity under off-Hartmann–Hahn conditions; HETMAT NOESY connectivities in the putative 3D structures of the 5_SL5b+c and 5_SL8 SARS-CoV-2 fragments; additional HETMAT vs HMQC-NOESY comparisons recorded at 600 MHz (PDF)

## Abbreviations

HETMAT NOESY: HETeronuclear MAgnetization Transfer Nuclear Overhauser Efffect SpectroscopY
CP: cross-polarization
SNR: signal-to-noise ratio.
