# Supplementary material for: 3D Heteronuclear Magnetization Transfers for the Establishment of Secondary Structures in SARS-CoV-2-Derived RNAs
Source: J Am Chem Soc. 2021 Mar 30;143(13):4942–8. doi: 10.1021/jacs.1c01914 (PMC8154514; doi:10.1021/jacs.1c01914)
Supplement: Supplementary file 1 — ja1c01914_si_001.pdf [file ja1c01914_si_001.pdf]

Supplementary Information for

3D Heteronuclear Magnetization Transfers for the establishment of secondary structures in SARS-CoV-2-derived RNAs

Jihyun Kim[1,#], Mihajlo Novakovic[1,#], Sundaresan Jayanthi[2], Adonis Lupulescu[3], Eriks Kupce[4], J. Tassilo Grün[5], Klara Mertinkus[5], Andreas Oxenfarth[5], Christian Richter[5], Robbin Schnieders[5], Julia Wirmer-Bartoschek[5], Harald Schwalbe[5], and Lucio Frydman[1]

[1]Department of Chemical and Biological Physics, Weizmann Institute of Science, Rehovot 7610001, Israel

[2]Department of Physics, Indian Institute of Space Science and Technology, Valiamala, Thiruvananthapuram 695 547, Kerala, India

[3]Nicolae Titulescu nr. 8, Turda, Jud. Cluj, Romania

[4]Bruker Ltd, Banner Lane, Coventry, UK

[5]Institute of Organic Chemistry and Chemical Biology, Center for Biomolecular Magnetic Resonance, Johann Wolfgang Goethe-University, D-60438 Frankfurt/Main, Germany

[#]Equal contributions

1) Experimental section

Sample preparation. ^{15}N-labeled 5_SL5b+c and 5_SL8 RNA samples were produced by T7 polymerase-based *in-vitro* transcription, as described in Ref 1. RNA purity was verified by denaturing PAA gel electrophoresis and homogenous folding was monitored by native PAA gel electrophoresis. The final concentration of the 5_SL5b+c and 5_SL8 samples in the NMR tubes were 0.7 and 0.8 mM, respectively.

NMR experiments. NMR experiments were conducted using either a 600 MHz, 14.1 T Bruker magnet equipped with an Avance III console and TCI prodigy probe; or 1 GHz, 23.5 T Bruker Avance Neo spectrometer equipped with a TCI cryoprobe. For 3D HETeronuclear Magnetization Transfer (HETMAT) NOESY experiments, ^{1}H and ^{15}N chemical shifts of RNA peaks were chosen from ^{15}N-^{1}H HSQC spectra and then encoded in the CP block for the selective inversion. The nutation frequency and duration of CP were chosen as 50 Hz and 14.5 ms for most of the resonances; 75 Hz and 10.4 ms were used for a few broader imino resonances. The mixing time and number of loops were optimized depending on the samples to achieve a maximum NOE. Spectra were acquired 480 or 512 scans depending on sample concentrations. Each spectrum from HETMAT NOESY experiments was apodized with QSINE window function and Fourier transformed using Topspin software (Bruker Biospin). To build up a 2D CP-based spectrum,

spectra were placed in a 2D plot according to the ^{15}N chemical shifts. For comparison, conventional 2D HMQC-NOESY experiments were performed with mixing times of 150 – 200 ms. A total acquisition time was set as the same as CP-based counterparts. As a result, both measurements were set identical except for their encoding and mixing principles. Supporting Table S1 summarizes the main parameters used in various HETMAT and conventional 2D HMQC-NOESY acquisitions presented in this study. Additional experimental details, including experimental set-up and pulse sequences for HETMAT's implementation, can be downloaded from https://www.weizmann.ac.il/chemphys/Frydman_group/software.

Supporting Table S1. Parameters used for HMQC-NOESY and HETMAT experiments on Covid-19 fragments presented in the main text.

RNA	5_SL5b+c		5_SL8b+c		5_SL8b+c	
Parameters	HMQC NOESY	HETMAT NOESY	HMQC NOESY	HETMAT NOESY	HMQC NOESY	HETMAT NOESY
Temperature (K)	283	283	283	283	298	298
SW (ppm)	23.8	23.8	23.8	23.8	23.8	23.8
TD	3072	3072	3072	3072	3072	3072
SW_1 (ppm)	24	/	24	/	24	/
TD_1	384	13	384	21	384	17
NS	28	512	32	480	32	512
DS	32	32	32	32	32	32
d_1 (s)	1.5	1.5	1.5	1.5	1.5	1.5
RG	101	101	101	101	101	101
O_1 (F_2, ppm)	4.697	4.697	4.694	4.694	4.698	4.698
O_1 (F_1, ppm)	116	116	116	116	116	116
d_8 (NOE, ms)*	175	a: 125<br>b: 30	150	a: 80<br>b: 50	200	a: 125<br>b: 50
n*<br>(number of loops)	/	a: 7<br>b: 20	/	a: 10<br>b: 17	/	a: 7<br>b: 17
RF CP field*<br>$\omega_1/2\pi$ (Hz)	/	a: 50<br>b: 75	/	a: 50<br>b: 75	/	a: 50<br>b: 75

* a and b indicate the different parameters applied for narrow and broad peaks, respectively.

2) Deriving the time evolution of an I_z state upon establishing a Hartmann-Hahn contact

The relevant Hamiltonian to analyse the narrowband longitudinal CP of the imino groups, is that of two *J*-coupled spin-half nuclei *I* and *S*, simultaneously subject to on-resonance RF irradiation. Assuming the latter is continuously applied along the *x*-axes of the doubly *I,S* rotating frame, this Hamiltonian is

$$H = \omega_{1I} I_x + \omega_{1S} S_x + 2\pi J_{IS} I_z S_z \quad \text{[S1]}$$

It is useful to analyse the associated spin dynamics in a tilted reference frame,[2] where the *z*-axis is now colinear with the RF fields

$$H' = e^{+i\frac{\pi}{2}(I_y+S_y)} H e^{-i\frac{\pi}{2}(I_y+S_y)} = \omega_{1I} I_z + \omega_{1S} S_z + 2\pi J_{IS} I_x S_x \quad \text{[S2]}$$

The initial density operator $\rho(0) = I_z$ becomes $-I_x$ in this frame; the state of the system after a time *t* of irradiation is thus described by

$$\rho(t) = e^{-i\frac{\pi}{2}(I_y+S_y)} e^{-iH't}(-I_x) e^{iH't} e^{i\frac{\pi}{2}(I_y+S_y)}. \quad \text{[S3]}$$

Evaluation of this state requires calculation of $e^{-iH't}$ and $\rho_{tilt}(t) = e^{-iH't}(-I_x)e^{iH't}$. Relabeling the Zeeman $|m_I m_S\rangle$ states as $|++\rangle = |1\rangle$; $|+-\rangle = |2\rangle$; $|-+\rangle = |3\rangle$; $|--\rangle = |4\rangle$, it is possible to write H' as the sum of two commuting parts, $H' = H'_{ZQ} + H'_{DQ}$, acting in the 2-3 (or zero-quantum) subspace and 1-4 (or double-quantum) subspaces respectively.[3] Moreover,

$$H'_{ZQ} = \Delta Z^{2-3} + \pi J_{IS} X^{2-3} \quad \text{[S4]}$$

$$H'_{DQ} = \sigma Z^{1-4} + \pi J_{IS} X^{1-4} \quad \text{[S5]}$$

where $\Delta = (\omega_{1I} - \omega_{1S})$, $\sigma = (\omega_{1I} + \omega_{1S})$ and Z^{p-q}, X^{p-q} are fictitious spin-half operators connected with states $|p\rangle$ and $|q\rangle$.[4] The propagator corresponding to H' can then be written as

$$U' = e^{-iH't} = U_{DQ} + U_{ZQ} \quad \text{[S6]}$$

Utilizing the spin-half identity $\exp(-i\boldsymbol{S}\cdot\hat{\boldsymbol{n}}\phi) = \cos(\phi/2)\mathbf{1} - 2i\boldsymbol{S}\cdot\hat{\boldsymbol{n}}\sin(\phi/2)$, we obtain

$$U_{ZQ} = cos\ \phi_\Delta/2 - 2isin\ (\phi_\Delta/2)(\cos\theta_\Delta Z_{23} + \sin\theta_\Delta X_{23}), \quad \text{[S7]}$$

$$U_{DQ} = cos\ (\phi_\sigma/2) - 2isin\ (\phi_\sigma/2)(\cos\theta_\sigma Z_{23} + \sin\theta_\sigma X_{23}), \quad \text{[S8]}$$

where $\phi_\Delta = \sqrt{\Delta^2 + \pi^2 J_{IS}^2}\, t, \phi_\sigma = \sqrt{\sigma^2 + \pi^2 J_{IS}^2}\, t,\ \tan\theta_\Delta = \pi J_{IS}/\Delta, \tan\theta_\sigma = \pi J_{IS}/\sigma$.

Taking into account that $\rho_{tilt}(0) = -I_x = -X^{1-3} - X^{2-4}$ and employing Eq. [S7-8] we obtain

$$\rho_{tilt}(t) = A|1\rangle\langle 3| + A|2\rangle\langle 4| + B|1\rangle\langle 2| - B|3\rangle\langle 4| + H.C. \quad \text{[S9]}$$

where

$$A = -\frac{1}{2}[(cos(\phi_\Delta/2) - isin(\phi_\Delta/2)\cos\theta_\Delta)(cos(\phi_\sigma/2) - isin(\phi_\sigma/2)\cos\theta_\sigma Z_{23}) + sin\,(\phi_\Delta/2)sin\,(\phi_\sigma/2)\sin\theta_\Delta\sin\theta_\sigma] \quad [S10]$$

$$B = -\frac{1}{2}[(cos(\phi_\Delta/2) + isin(\phi_\Delta/2)\cos\theta_\Delta)(-isin(\phi_\sigma/2)\sin\theta_\sigma) - isin(\phi_\Delta/2)\sin\theta_\Delta(cos\,(\phi_\sigma/2) - isin\,(\phi_\sigma/2)\cos\theta_\sigma)] \quad [S11]$$

Expressing operators $|1\rangle\langle 3|$, $|1\rangle\langle 2|$, etc. in Eq. [S9] in terms of products of Cartesian spin operators and passing back to the normal rotating frame we obtain

$$\rho(t) = A(-I_z + iI_y) + 2BI_x(-S_z + iS_y) + H.C. \quad [S12]$$

where *H. C.* denotes the Hermitian conjugate terms. At Hartmann–Hahn match ($\omega_{1I} = \omega_{1S} \rightarrow \sigma = 2\omega_1, \Delta = 0$), the *A* and *B* terms in Eq. [S12] simplify considerably, and this becomes

$$\rho(t) = c_Z(t)\boldsymbol{I_z} + c_Y(t)\boldsymbol{I_y} + c_{XY}(t)\boldsymbol{I_x S_y} + c_{XZ}(t)\boldsymbol{I_x S_z} \quad [S13]$$

where

$$c_Z(t) = cos\left(\frac{\pi J_{IS}t}{2}\right)cos\left(\sqrt{4\omega_1^2 + \pi^2 J_{IS}^2}\,\frac{t}{2}\right) + sin\left(\frac{\pi J_{IS}t}{2}\right)sin\left(\sqrt{4\omega_1^2 + \pi^2 J_{IS}^2}\,\frac{t}{2}\right)\sin\theta_\sigma \quad [S14]$$

$$c_Y(t) = -\cos\theta_\sigma cos\left(\frac{\pi J_{IS}t}{2}\right)sin\left(\sqrt{4\omega_1^2 + \pi^2 J_{IS}^2}\,\frac{t}{2}\right) \quad [S15]$$

$$c_{XZ}(t) = 2\cos\theta_\sigma sin\left(\frac{\pi J_{IS}t}{2}\right)sin\left(\sqrt{4\omega_1^2 + \pi^2 J_{IS}^2}\,\frac{t}{2}\right) \quad [S16]$$

$$c_{XY}(t) = -2\sin\theta_\sigma cos\left(\frac{\pi J_{IS}t}{2}\right)sin\left(\sqrt{4\omega_1^2 + \pi^2 J_{IS}^2}\,\frac{t}{2}\right) + 2sin\left(\frac{\pi J_{IS}t}{2}\right)cos\left(\sqrt{4\omega_1^2 + \pi^2 J_{IS}^2}\,\frac{t}{2}\right) \quad [S17]$$

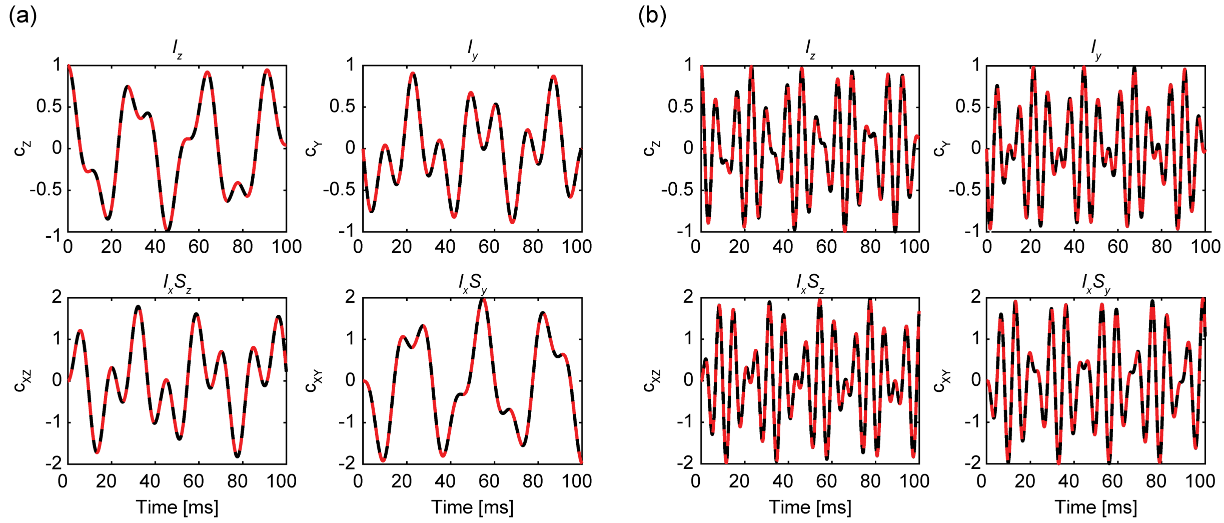

Figure S1. Comparisons between the spin dynamics upon irradiating a weak CP simulated using two-spin density matrix (black) and calculated using theorical expressions shown in Eqs. [S14]-[S17] (red) when CP is applied with nutation frequencies of (a) 50 Hz and (b) 150 Hz.

and

$$\cos\theta_\sigma = 2\omega_1/\sqrt{4\omega_1^2 + \pi^2 J_{IS}^2}, \qquad \sin\theta_\sigma = \pi J_{IS}/\sqrt{4\omega_1^2 + \pi^2 J_{IS}^2}. \qquad [S18]$$

Equation [S14] corresponds to Eq. (2) in the main manuscript. Figure S1 compares how good do the analytical predictions of Eqs. [S14]-[S18] compare with brute-force propagations of a two-spin density matrix driven by the Hamiltonian in Eq. [S1]; as can be appreciated, the agreement is excellent.

HETMAT NOESY correlations also rely on a reference scan, where RF is only irradiated on the I spin (the off-resonance scan in the experiment). In this case, the coefficient of I_z becomes

$$c_Z^{off} = \frac{\pi^2 J_{IS}^2 + \omega_1^2 cos\left(\sqrt{\omega_1^2+\pi^2 J_{IS}^2}\, t\right)}{\omega_1^2+\pi^2 J_{IS}^2} \qquad [S19]$$

which is Eq. (3) in the main text. The difference between the two coefficients defining the experiment's contrast, $\left|c_Z - c_Z^{off}\right|$, is

$$c_Z^{on} - c_Z^{off} = cos\left(\frac{\pi J_{IS} t}{2}\right) cos\left(\omega_{eff}^{IS}\ \frac{t}{2}\right) + sin\left(\frac{\pi J_{IS} t}{2}\right) sin\left(\omega_{eff}^{IS}\ \frac{t}{2}\right)\left(\frac{\pi J_{IS}}{\omega_{eff}^{IS}}\right) - \frac{\pi^2 J_{IS}^2 + \omega_1^2 cos\left(\omega_{eff}^{I}\, t\right)}{(\omega_{eff}^{I})^2}$$

[S20]

where $\omega_{eff}^{IS} = \sqrt{4\omega_1^2 + \pi^2 J_{IS}^2}$ and $\omega_{eff}^{I} = \sqrt{\omega_1^2 + \pi^2 J_{IS}^2}$ [S21]

If one can assume that the ω_1 is larger than $2J_{IS}$, the expression for this difference can be approximated as

$$c_z^{on} - c_z^{off} = cos\left(\frac{\pi J_{IS} t}{2}\right) cos(\omega_1 t) + sin\left(\frac{\pi J_{IS} t}{2}\right) sin(\omega_1 t)\left(\frac{\pi J_{IS}}{2\omega_{1I}}\right) - cos(\omega_1 t) \qquad [S22]$$

Calculating the derivative of this equation and setting it to zero to find the maximal on/off contrast, requires making

$$\sin(\omega_1 t)\,[1 - cos(\pi J_{IS} t/2)] = 0 \qquad [S23]$$

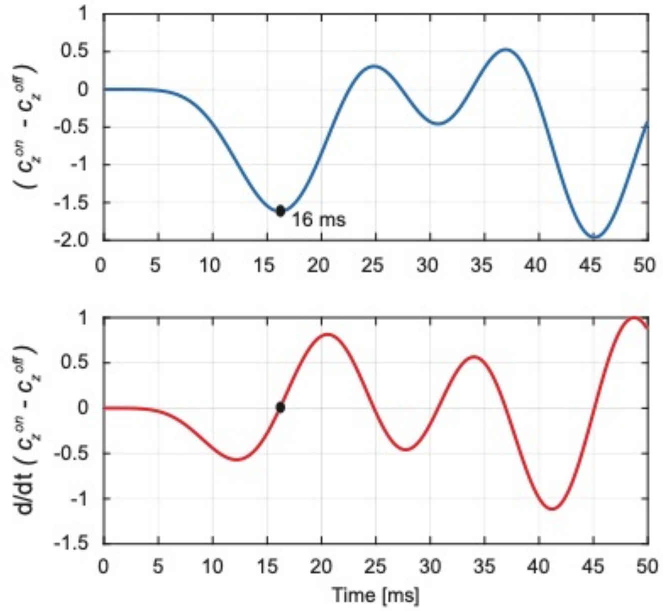

Figure S2. The difference between c_z coefficients with on- and off-resonance CP calculated from the equation S20 (blue) and its derivative (red). Parameters for the calculation are $\omega_{1H}/2\pi = \omega_{1N}/2\pi = 50\ Hz$ and $J_{NH} = 90\ Hz$. Note that the first minimum of the difference is when CP mixing time is about 16 ms, showing in good agreement with the value experimentally determined (black dots, see Figure S3).

Eq. [S23] has many local minima and maxima. From Eq. [S22] it can be seen that the first global maximum/minimum should occur at a time $t_{opt} \cong 2/J_{IS}$, for which $cos(\omega_1 t) = \pm 1$. This is easily achieved, since in Eq. [S22] RF terms oscillate rapidly in comparison with the J-coupling term. In the actual experiments we carried out, however, ω_1 is usually smaller than J_{IS}; the exact expression shown in Eq. [S20] should be used. An analysis of the optimal ensuing time is thus analyzed in Figure S2, which plots both $(c_z^{on} - c_z^{off})$ and its derivative vs CP contact time. Notice the good agreement between the analytical calculations, and the condition experimentally found for the RNA fragments (black dot).

3) Cross-polarization on- and off- the Hartmann-Hahn conditions: H_z inversion efficiency and ^{15}N-^{1}H spectral selectivity

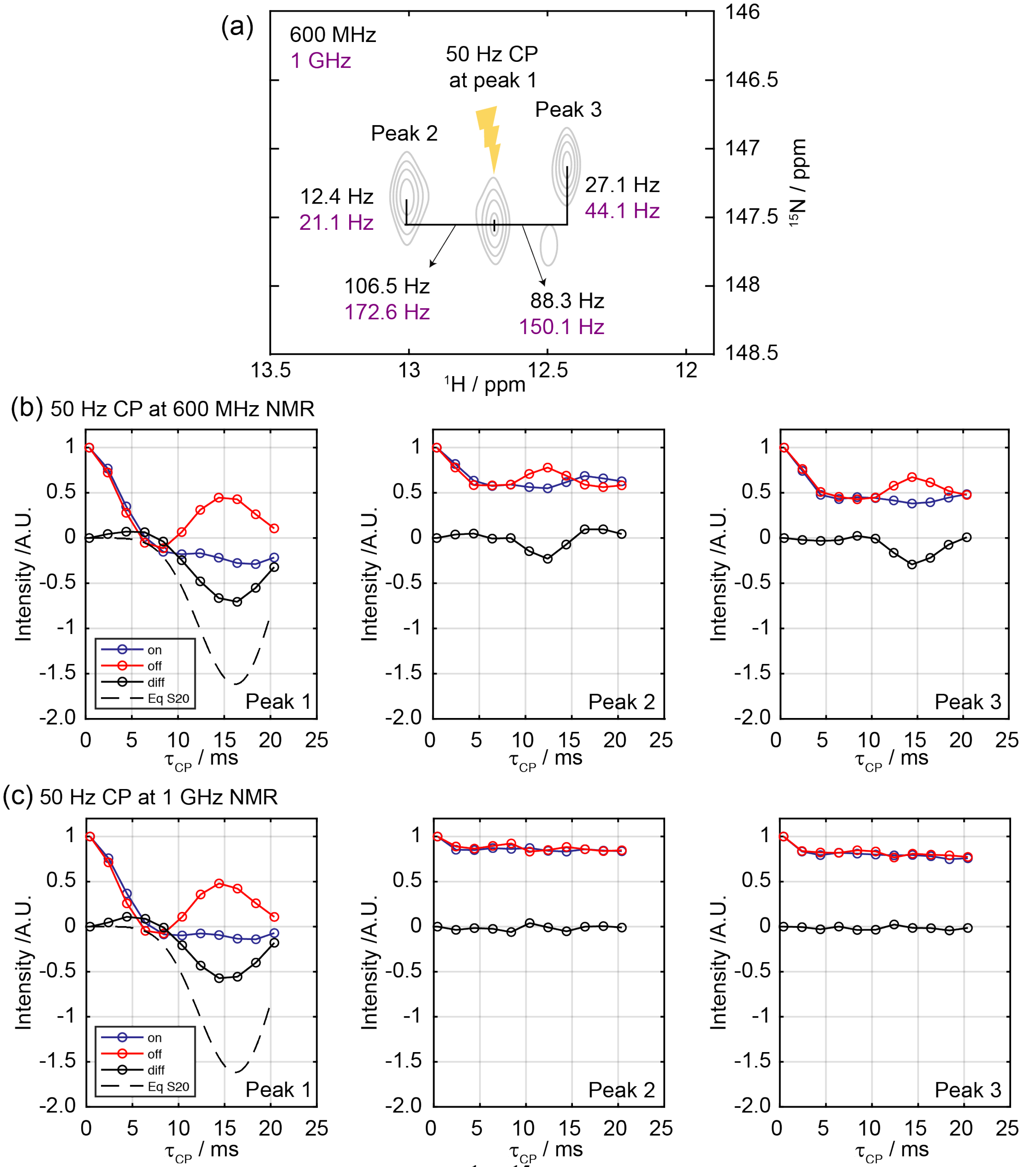

Figure S3. Narrowband CP nutation profiles for a ^{1}H-^{15}N spin pair on the 5_SL8 RNA sample, showing the achievable pseudo-2D selectivity. (a) A CP with matched RF frequencies of 50 Hz is applied at the peak at 12.7/147.6 ppm (on-resonance with peak 1), or at 12.7/154 ppm (for the off-resonance measurement). Distances between peak 1 and neighboring peaks are indicated in the figure. CP spectra were acquired with the sequence shown in Figure 1b (with N=1, τ_{NOE}=0 s) at (b) 600 MHz and (c) 1 GHz. Peak intensity changes are plotted when CP is on-resonance (blue), off-resonance (red), and its difference (black) as a function of CP duration for peak 1 (left column), peak 2 (middle column), and peak 3 (right column). Dashed lines indicate the predictions of the analytical derivation above. Note that the difference of the minimum value between experimental and analytical curves (black solid and dashed lines) may be due to the relaxation during the CP duration, which is not considered in the theorical treatment.

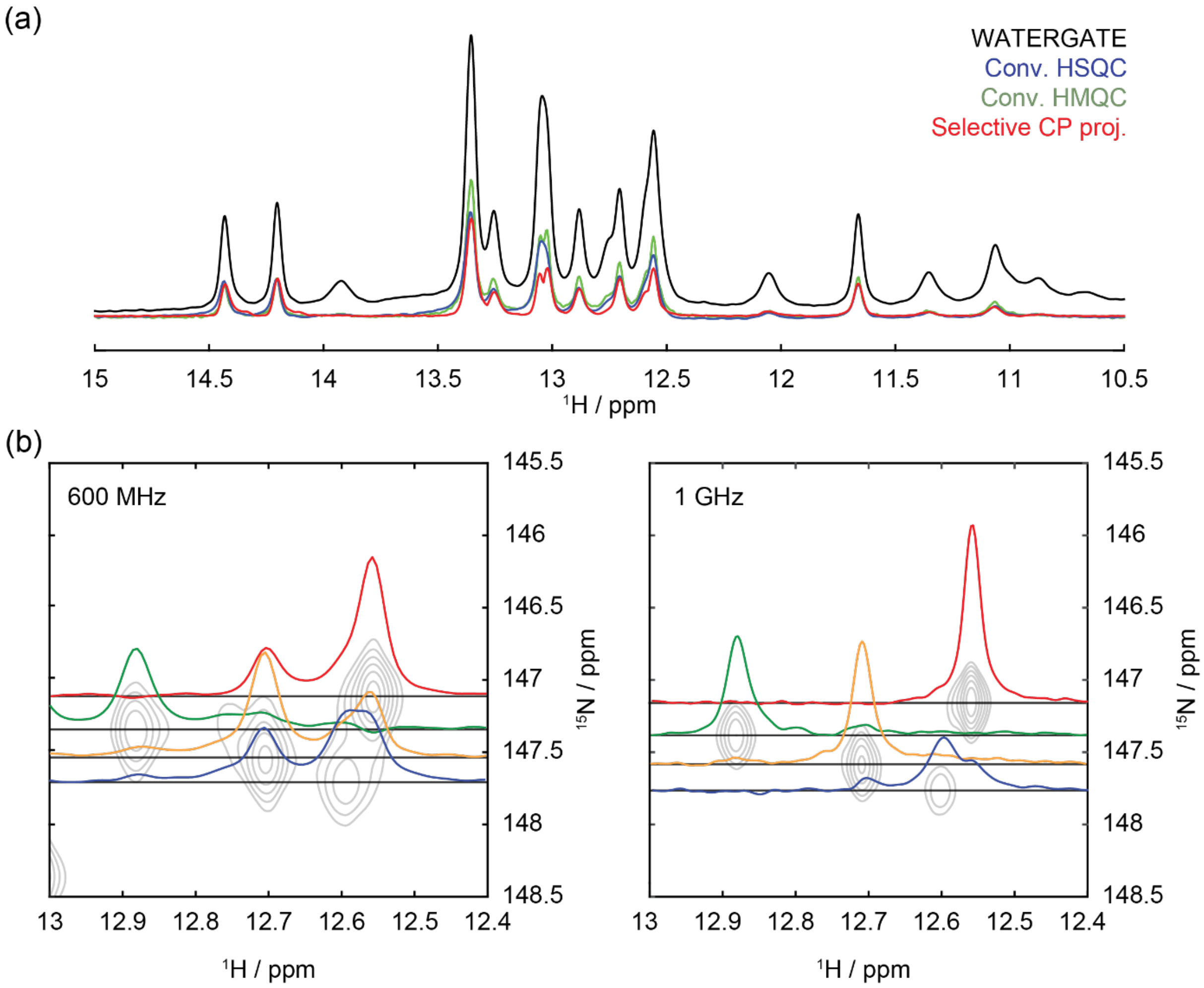

Figure S4. (a) Assessing the sensitivity of selective CP editing, by comparing 1D spectra measured using WATERGATE (black), conventional HSQC (blue), conventional HMQC (green), and the projection of the HETMAT spectrum (red) acquired using the pulse sequence shown in Figure 1b with N=1, τ_{NOE}=0 s is shown for 5_SL8. (b) Testing selective CP's ability to resolve four closely spaced resonances: 1D slices of the four selected peaks are overlaid on conventional 2D HSQCs of 5_SL8 recorded at 600 MHz (left) and 1 GHz NMR. Note how the selectivity of CP benefits from operating at 1 GHz.

4) HETMAT vs HMQC-NOESY: Sensitivity comparisons on SARS-CoV-2 fragments

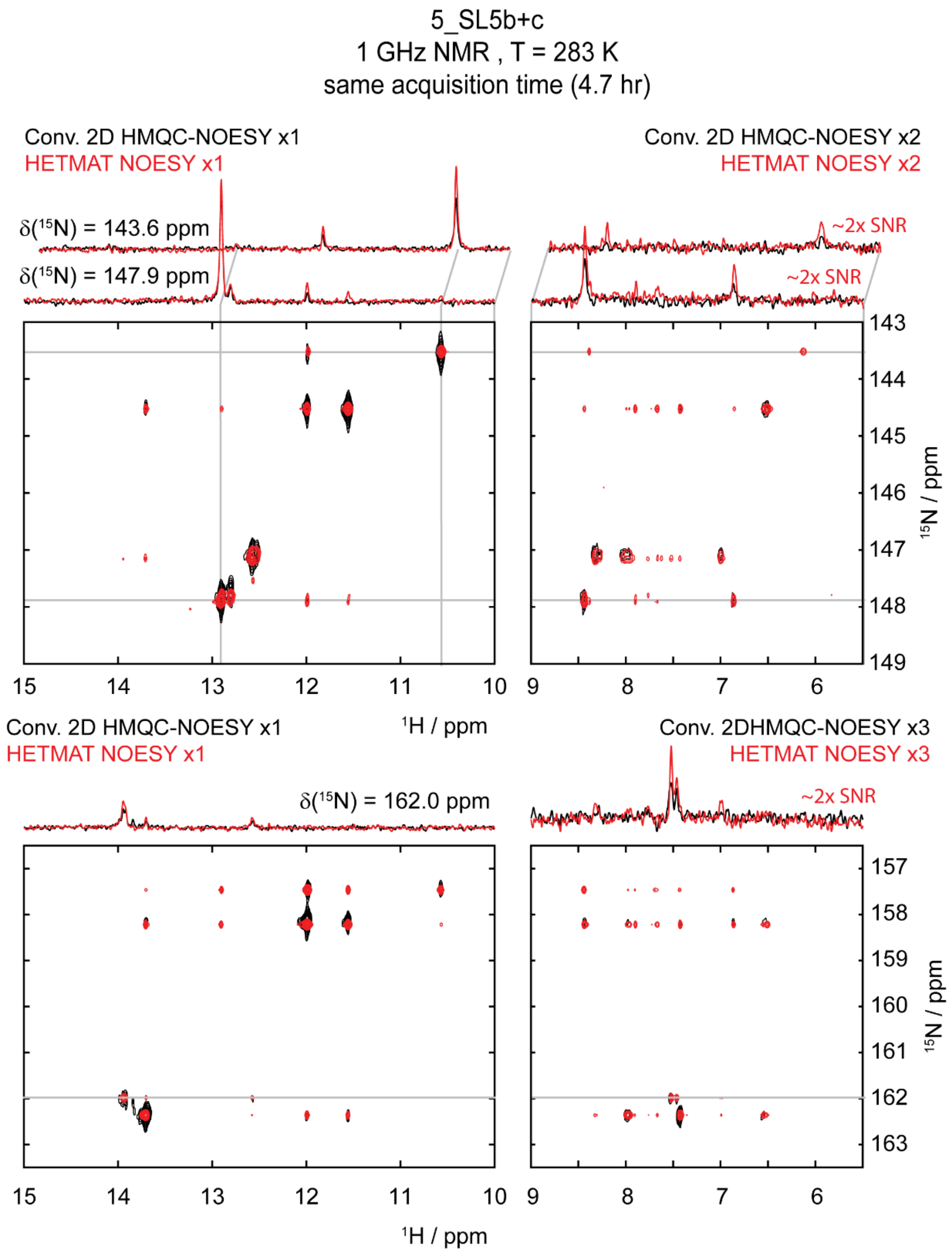

Figure S5. Comparison between conventional HMQC-NOESY (black) and HETMAT NOESY (red) spectra acquired on 5_SL5b+c RNA fragment measured using 1 GHz NMR at 283 K. Conditions were as shown in Figure 2 of the main text, but here both spectra are shown overlaid on one another. 1D slices at selected ^{15}N chemical shifts are shown above each panel.

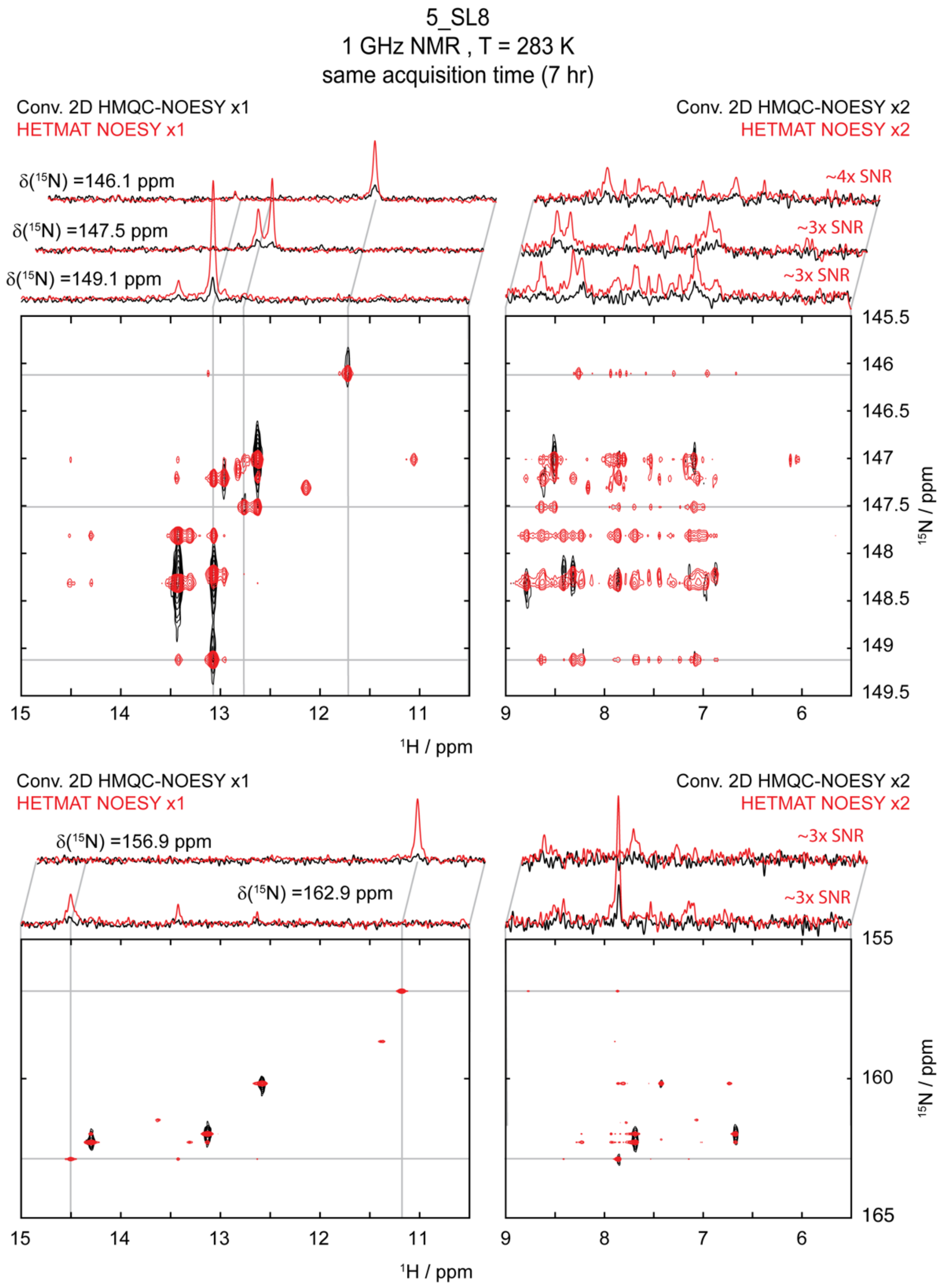

Figure S6. Idem as Figure S5, but for the 5_SL8 RNA fragment. Acquisition parameters for these data are as detailed in Figure 3 of the main text.

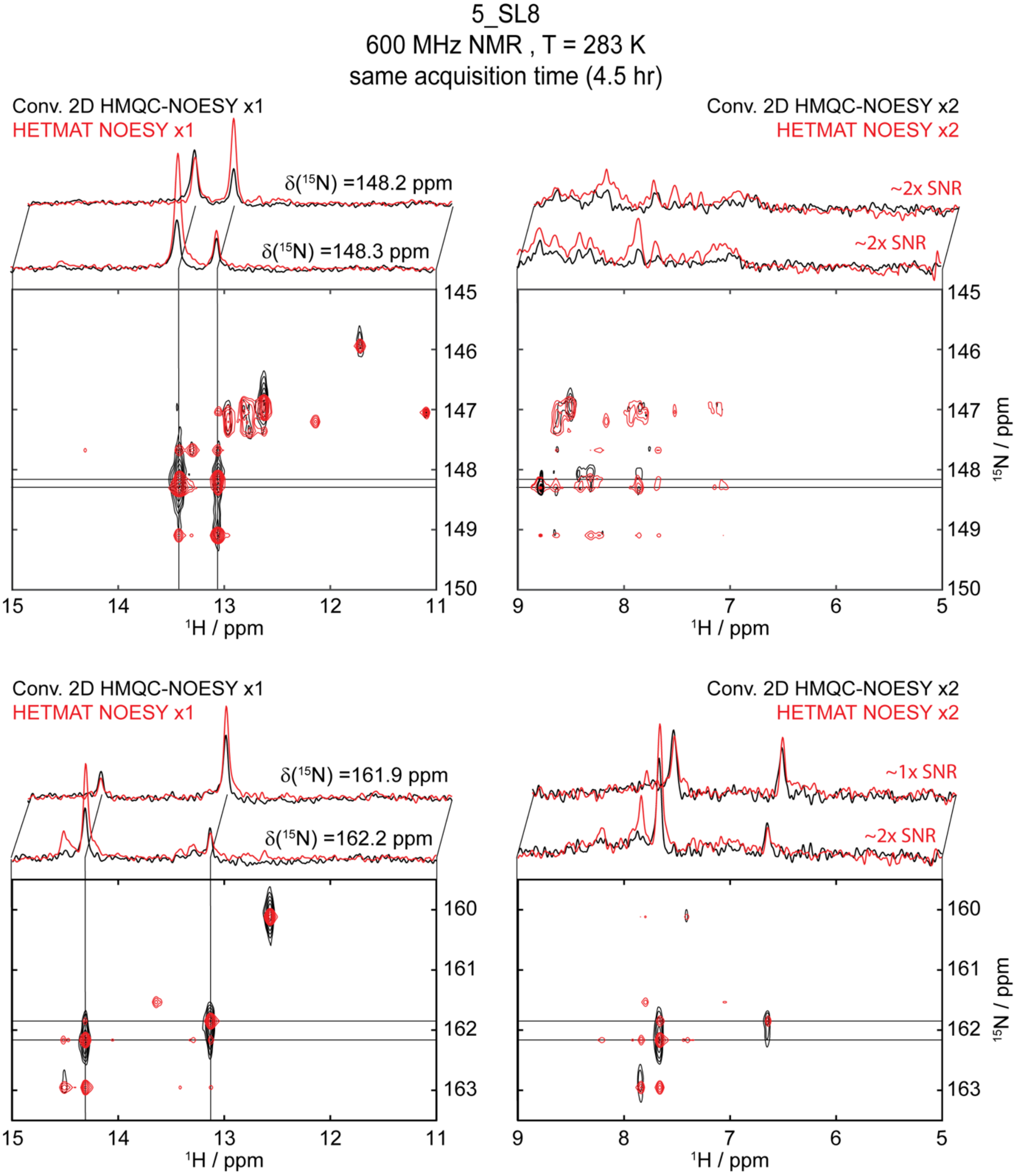

Figure S7. Idem as Figure S6, but for experiments collected at 600 MHz. Notice the cross-talk between peaks at 148.2 and 148.3 ppm (top), absent at higher fields.

5) Improving HETMAT's spectral selectivity by cross-peak discriminations

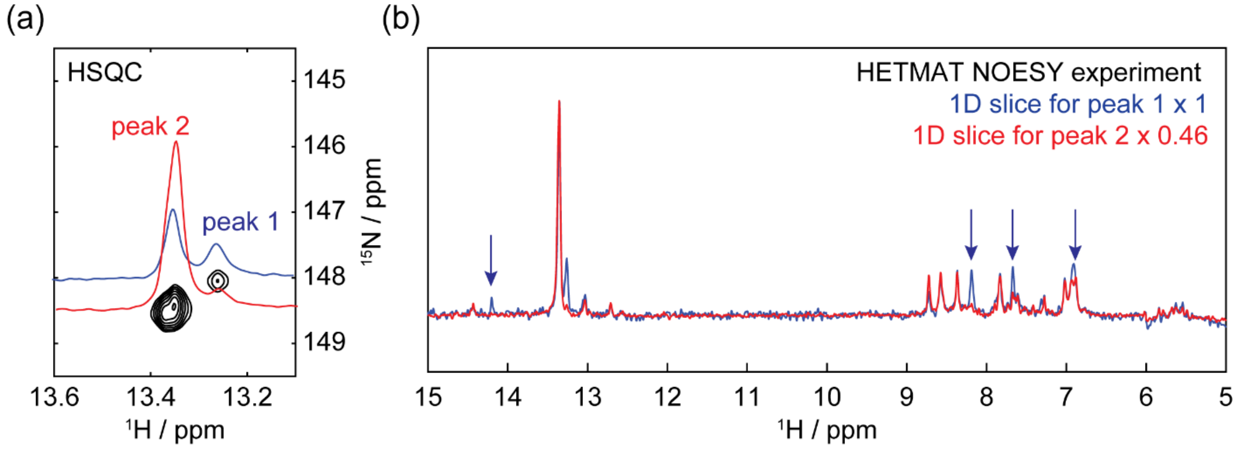

Figure S8. (a) Illustrating HETMAT's spectral selectivity problems for 5_SL8: upon performing the narrowband CP inversion of peak 1 (blue trace), a simultaneous substantial inversion of peak 2 becomes evident in a subsequent HSQC acquisition. Nevertheless, the opposite is not true: CP selection of peak 2 does not appreciably label the broader peak 1. (b) Utilizing this asymmetry to discriminate the NOE cross-peaks originating from each of the residues (cross-peaks originating from peak 1 are marked with blue arrows), and can be identified from the NOE traces arising from the HETMAT encoding for each peak.

6) HETMAT vs HMQC-NOESY performances at room temperature

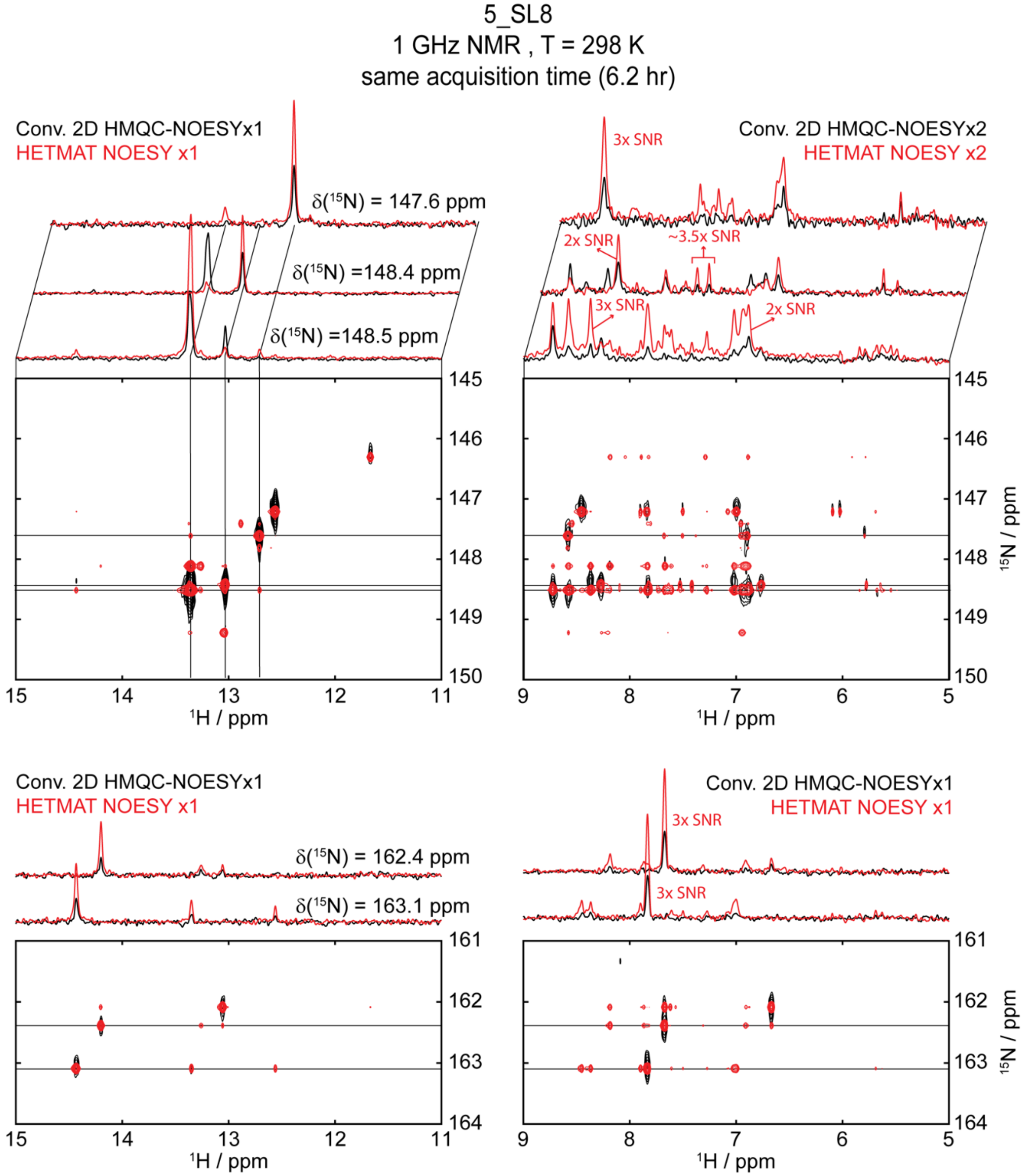

Figure S9. Same as in Figure S6, but measured at 298 K. Both sets of experiments were performed in the same experimental time; the conventional spectrum was acquired with 200 ms mixing, while HETMAT was collected with either 7x125 ms mixing loops or 17x 50 ms mixing loops (depending on the broadeness of the imino proton peaks). 1D slices at selected ^{15}N chemical shifts are shown on top of each panel.

7) Exemplifying HETMAT's improved sensitivity under off-Hartmann-Hahn conditions

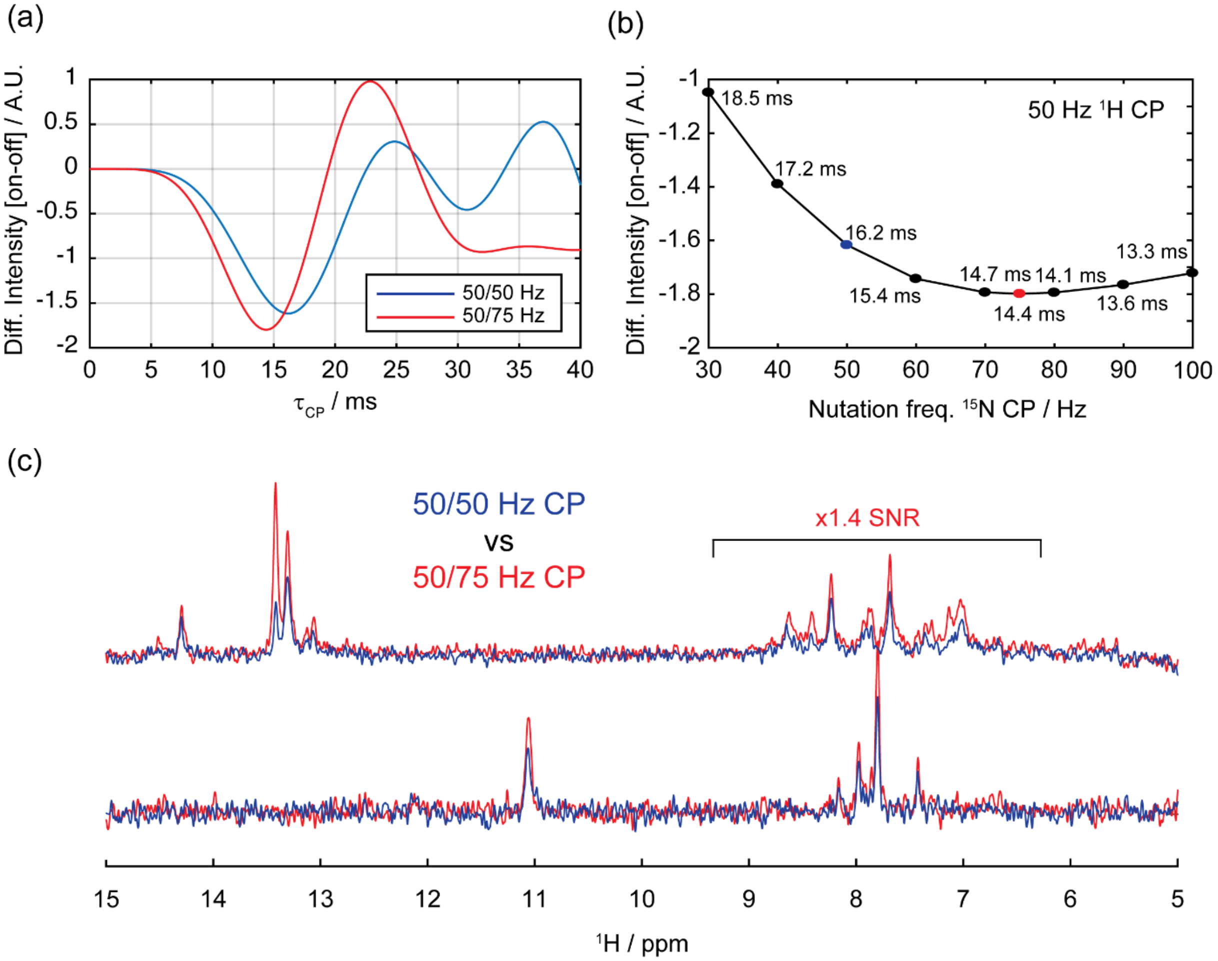

Figure S10. (a) Simulated profiles of selective CP of ^{1}H-^{15}N spin pair with 50 Hz RF fields applied on both channels (blue), and with $\omega_1/2\pi$ = 50/75 Hz for ^{1}H/^{15}N (red). (b) Maximizing (in absolute value) the differences in intensity between on- and off-resonance CP as a function of nutation frequency of ^{15}N CP; the nutation frequency of the ^{1}H was fixed at 50 Hz during these simulations, and the t_{opt} durations for each case are labeled. (c) Comparisons of HETMAT spectra recorded under matched (blue) and mismatched (red) Hartmann-Hahn, using the indicated ^{1}H and ^{15}N RF fields.

7) HETMAT NOESY connectivities in the putative 3D structures of the 5_SL5b+c and 5_SL8 SARS-CoV-2 fragments

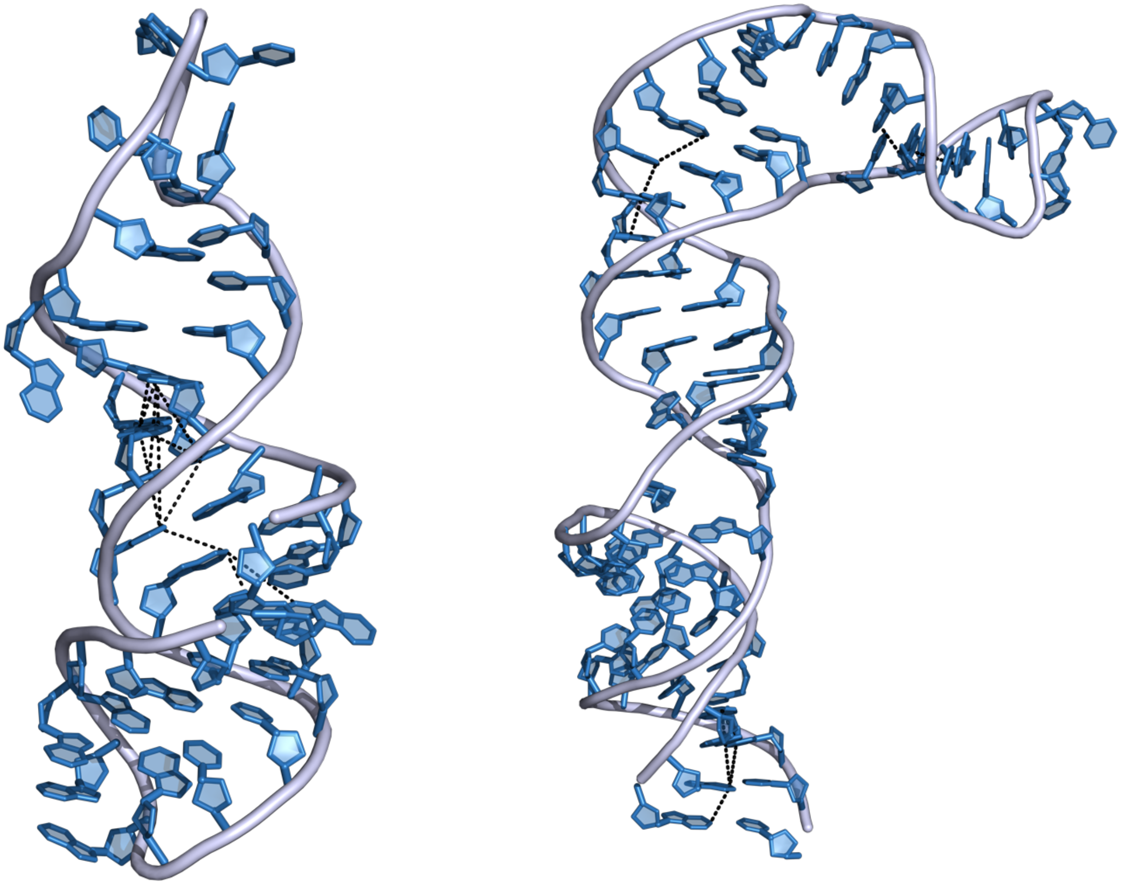

Figure S11. 3D models of 5_SL5bc (left) and 5_SL8 (right) generated from the secondary structures proposed by Wacker et al,[1] using RNAComposer.[5,6] Black dashed lines indicate the imino-to-imino NOE correlations that were observed and unambiguously assigned for each fragment using HETMAT NOESY 1GHz experiments; several cross-peaks for the 5_SL8 case remain to be assigned.

References

(1) A. Wacker *et al*. Secondary Structure Determination of Conserved SARS-CoV-2 RNA Elements by NMR Spectroscopy. *Nucleic Acids Res.* **2020**, *48*, 12415.

(2) D. E. Demco, J. Tegenfeldt, and J. S. Waugh, Dynamics of cross relaxation in nuclear magnetic double resonance , *Phys. Rev. B* **1975,** *11*, 4133.

(3) M. H. Levitt, D. Suter, and R. R. Ernst, Spin dynamics and thermodynamics in solid-state NMR cross polarization, *J. Chem. Phys.* **1986** 84, 4243.

(4) S. Vega, Fictitious spin 1/2 operator formalism for multiple quantum NMR*, J. Chem. Phys.* **1978** 68, 5518.

(5) M. Popenda, M. Szachniuk, M. Antczak, K. J. Purzycka, P. Lukasiak, N. Bartol, J. Blazewicz, R. W. Adamiak, Nucleic Acids Res. **2012**, 40, 1.

(6) M. Antczak, M. Popenda, T. Zok, J. Sarzynska, T. Ratajczak, K. Tomczyk, R. W. Adamiak, M. Szachniuk, Acta Biochim. Pol. **2016**, 63, 737.
